# Single-Molecule/Cell Analyses Reveal Principles of Genome-Folding Mechanisms in the Three Domains of Life

**DOI:** 10.3390/ijms222413432

**Published:** 2021-12-14

**Authors:** Hugo Maruyama, Takayuki Nambu, Chiho Mashimo, Toshinori Okinaga, Kunio Takeyasu

**Affiliations:** 1 Department of Bacteriology, Osaka Dental University, Hirakata 573-1121, Japan; nambu-t@cc.osaka-dent.ac.jp (T.N.); mashimo@cc.osaka-dent.ac.jp (C.M.); okinaga@cc.osaka-dent.ac.jp (T.O.); 2 Graduate School of Biostudies, Kyoto University, Kyoto 606-8501, Japan; takeyasu@lif.kyoto-u.ac.jp; 3 Center for Biotechnology, National Taiwan University, Taipei 10672, Taiwan

**Keywords:** comparative structural/molecular biology, higher-order chromosome structure, nucleoid, atomic force microscopy, Hi-C, mass spectrometry, horizontal gene transfer

## Abstract

Comparative structural/molecular biology by single-molecule analyses combined with single-cell dissection, mass spectroscopy, and biochemical reconstitution have been powerful tools for elucidating the mechanisms underlying genome DNA folding. All genomes in the three domains of life undergo stepwise folding from DNA to 30–40 nm fibers. Major protein players are histone (Eukarya and Archaea), Alba (Archaea), and HU (Bacteria) for fundamental structural units of the genome. In Euryarchaeota, a major archaeal phylum, either histone or HTa (the bacterial HU homolog) were found to wrap DNA. This finding divides archaea into two groups: those that use DNA-wrapping as the fundamental step in genome folding and those that do not. Archaeal transcription factor-like protein TrmBL2 has been suggested to be involved in genome folding and repression of horizontally acquired genes, similar to bacterial H-NS protein. Evolutionarily divergent SMC proteins contribute to the establishment of higher-order structures. Recent results are presented, including the use of Hi-C technology to reveal that archaeal SMC proteins are involved in higher-order genome folding, and the use of single-molecule tracking to reveal the detailed functions of bacterial and eukaryotic SMC proteins. Here, we highlight the similarities and differences in the DNA-folding mechanisms in the three domains of life.

## 1. Overview of Genome-Folding Mechanisms

**Figure 1:**
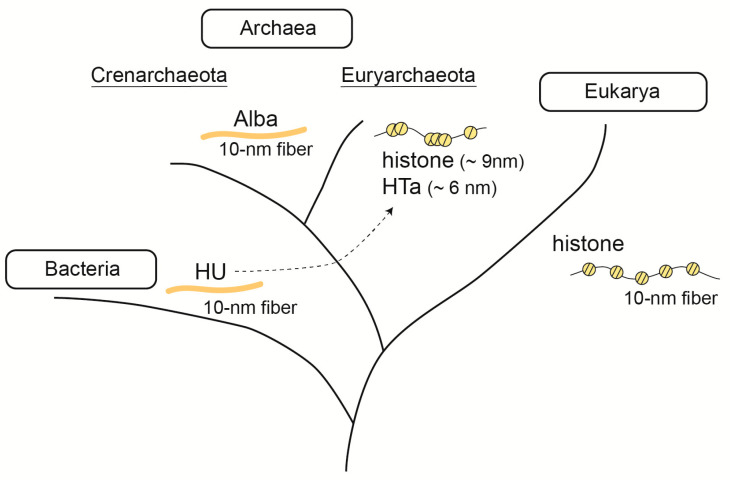
Initial steps of genome folding in the three domains of life. In Eukarya, the nucleosome formed by histone is the fundamental unit of genome structure. In bacteria, the conserved protein HU forms a fibrous structure on DNA. In archaea, euryarchaeota encodes DNA-wrapping proteins, histone or HTa. The archaeal histone forms “hypernucleosome”, which consist of varying numbers of histone dimers. HTa have horizontally transferred from bacteria to archaea at some point. Alba is widely distributed among archaea and is capable of forming fibrous structures.

Genomic DNA must be properly folded inside the cell to simultaneously achieve compaction and relaxation (for transcription and replication) at the same time in all living organisms. Chromosome architectures in the three domains—Eukarya, Bacteria, and Archaea—are different in their protein components, but similar at the nanometer scale at both fundamental and higher-order structure levels (Figure 1).

In eukaryotes, the most fundamental structural unit of chromosomes is the nucleosome. In a nucleosome, approximately 146–147 bp of double-stranded DNA is wrapped around a histone octamer, which is composed of a core (H3/H4)_2_ tetramer and two H2A/H2B dimers [1,2]. The diameter of the nucleosome is approximately 11 nm. With the help of nucleosome remodeling complexes, nucleosomes are spaced regularly, resulting in a so called ”beads-on-a-string” structure; digestion of this structure with micrococcal nuclease (MNase) results in a ladder-like pattern upon agarose gel electrophoresis [3]. The beads-on-a-string structure is further folded into a thicker ~30 nm fiber structure with the help of linker histone H1 and other proteins [4]. Eukaryotic chromosomes are organized into either tightly packed, transcriptionally inactive heterochromatin, or loosely packed, transcriptionally active euchromatin. There are different molecular complexes in these chromatin regions with different transcriptional activity [5]. The state that a given region of chromosomes will be in depends on the types of cells; if the expression of genes in a given chromosomal region is not required for the state of the cell, the region is likely to be packed into heterochromatin [6]. Further condensation of chromosomes occurs during the mitotic phase of the cell cycle; the structural maintenance of chromosomes (SMC) complex is responsible for this condensation [7]. The local and higher-order structures of eukaryotic chromosomes have been studied using a variety of techniques, including sequencing-based and microscopy-based techniques [8,9].

In bacteria, genomic DNA is folded into nucleoids through the aid of nucleoid-associated proteins (NAPs). Heat-unstable nucleoid protein (HU) is a major NAP that is widely distributed among bacteria; at least one HU subunit is present in most bacterial genomes [10]. Other NAPs are more specific to certain bacterial lineages. For example, Gram-negative bacteria (e.g., *Escherichia coli*) encode factor for inversion stimulation (Fis), integration host factor protein (IHF), host factor for phage Qbeta RNA replication (Hfq), histone-like nucleoid structuring protein (H-NS) and its paralog suppressor of T4 *Td* mutant phenotype A (StpA), and DNA-binding protein from starved cells (Dps) [10]. Among these NAPs, H-NS is highly conserved within *E. coli* and related strains, and Fis is conserved among Enterobacteriaceae [11]. These small proteins can fold DNA by bending, wrapping, or bridging double-stranded DNA strands [10].

Structural studies have shown that *E. coli* nucleoids form a hierarchical structure consisting of 10, 30, and 80 nm fibers and 300 nm loops [12]. RNase digestion showed that RNA molecules are involved in the folding of fibers from 10 to 30 nm and 30 to 80 nm, and that NAPs are involved in all folding steps [12]. The chloroplast, which is a eukaryotic organelle that has evolved from endosymbiotic cyanobacteria, has a nucleoid consisting of 20, 30, 40, and 70–80 nm fibers [13]. Thus, similar to eukaryotic chromosomes, bacterial nucleoids are folded hierarchically.

The structure of bacterial nucleoids changes dynamically during growth phase progression. The expression of Dps of *E. coli* protein increases towards the stationary phase, thereby contributing to nucleoid compaction [14,15]. The number of other NAPs is usually higher in the exponential phase than in the stationary phase. Single-genome analysis by atomic force microscopy (AFM) has revealed that oxidative stress triggers metallo-regulon gene A (MrgA; a staphylococcal Dps homolog)-dependent nucleoid compaction in *S. aureus* [16].

NAPs are associated with bacterial pathogenicity. For example, NAPs, including HU, H-NS, Fis, and IHF, affect the expression of virulence genes in *Salmonella enterica* serovar Typhimurium [17]. HU is involved in the viability of the pneumonia pathogen *Streptococcus pneumoniae* [18] and is involved in the expression of surface polysaccharides, an important virulence determinant for the periodontal pathogen *Porphyromonas gingivalis* [19]. It also regulates genes involved in acid adaptation in *Helicobacter pylori* [20].

Archaea is a domain of life, along with Eukarya and Bacteria [21]. Although archaeal species are morphologically similar to bacterial species, there are fundamental differences between the two domains, for example, the lipid component in the cell membrane, the structure of RNA polymerase, and the replication machinery [22]. Archaeal species live in extreme environments, such as high temperature, high acidity, and high salinity, as well as in milder environments such as soil, oceans, and the human body [23]. An increasing number of archaeal genomes have been found in environmental metagenomic studies, and archaea are considered to play important roles in mediating an array of ecological processes, including global carbon and nutrient cycles [22]. Several archaeal species have been reported to be associated with human diseases such as periodontal disease and inflammatory bowel disease [24]. Therefore, it is becoming increasingly important to understand how archaea respond to and influence their environment through the control of gene expression.

Archaea have a eukaryotic-like basal transcription apparatus, but their gene expression is mainly controlled by bacterial-like transcription factors [25,26]. Growing evidence suggests, however, that it is also regulated at the level of higher-order chromosome structure, similar to the case of eukaryotes and bacteria. For example, the discovery of a global transcriptional repressor (TrmBL2 in *Thermococcales*) that binds to the entire genome without sequence specificity implies a link between higher-order chromosome architecture and gene expression [27,28], and recent Hi-C studies provide more direct evidence on the relationship between three-dimensional chromosome compartmentalization and transcriptional activity [29]. 

Archaea can be divided into two major groups based on the proteins they use to build their chromosomes. The majority of species in the phylum Euryarchaeota encode proteins homologous to eukaryotic histones, while those in Crenarchaeota use other DNA-binding proteins such as Alba and Cren7 [28]. Although our understanding of archaeal chromosome structure has greatly improved, it is still the most unexplored among the three domains [30]. Structural studies using AFM show that archaeal chromosomes have a hierarchically folded structure consisting of a 10 nm fibrous structure and 30–40 nm globular structures, regardless of the basic DNA-binding protein encoded.

In this review, we provide an overview of what we have learned to date about the chromosome structure across the three domains, focusing on the similarities and differences in the principle of their chromosome organization. First, we outline strategies to study chromosome structures. We then try to illustrate the principle of chromosome organization in the three domains.

## 2. Strategies to Study Chromosome Architecture and Function

**Figure 2:**
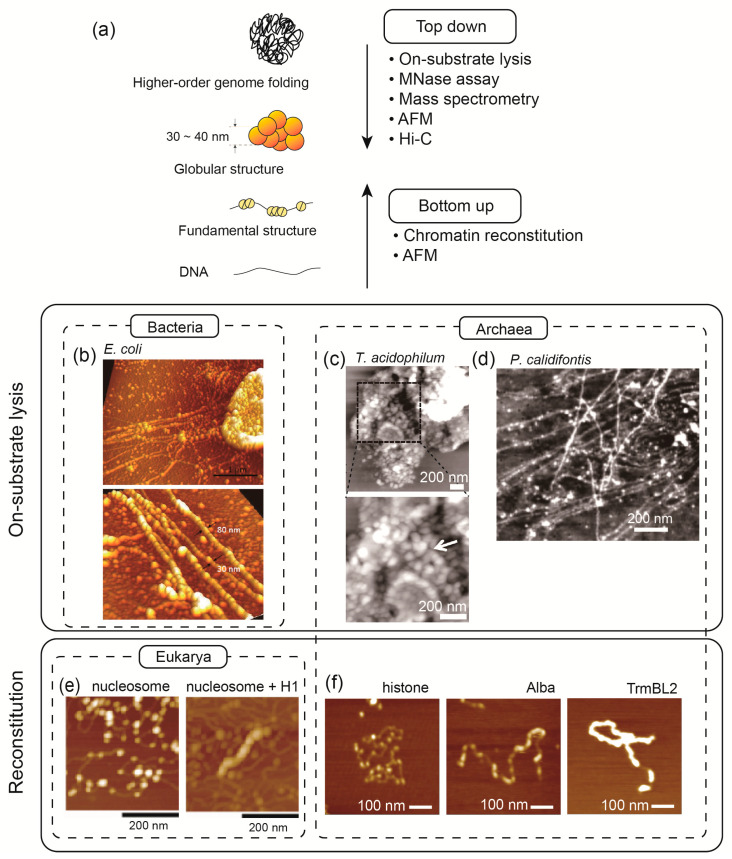
Methods for studying the structure of chromosomes. (**a**) The structural analyses of archaeal chromosome are shown as an example. Top-down approaches include on-substrate lysis, Micrococcal nuclease assay, mass spectrometry, AFM, and Hi-C, which are useful for identifying proteins responsible for higher-order genome folding as well as medium-scale chromosome folds such as 30–40 nm globular structure found in some archaea [31] and bacteria [15]. Bottom-up approaches include chromatin reconstitution in combination with AFM, are effective in identifying fundamental structural units and the factors required to fold them into higher-order structures. (**b**–**f**) Example AFM images taken in various approaches. On substrate lysis exhibits 30 and 80 nm nucleoid fiber from *E. coli* cell (**b**) [15], 30–40 nm globular structures from *T. acidophilum* cells (arrow) (**c**), and ~10 nm fibrous structure from *P. calidifontis* cells (**d**) [31]. (**e**) Reconstituted chromatin structure formed on DNA with eukaryotic histone octamer (left); a 30 nm fiber is formed with further addition of linker histone H1 (right) [32]. (**f**) Reconstituted archaeal chromosome structures formed with *T. kodakarensis* histone, Alba, and TrmBL2 [27].

To understand the hierarchical structure of chromosomes and their functions, it is essential to combine different levels of analysis. The structure of chromosomes or nucleoids from the fundamental unit to higher-order structures was uncovered by combining various approaches (Figure 2). These approaches can be categorized into “top-down” and “bottom-up” approaches. The top-down approaches aim to start from intact cells, then lyse the cells to analyze the exposed chromosome structure, trying to understand how the chromosomes are folded in vivo. On the other hand, bottom-up approaches analyze how chromosomes are constructed at the fundamental level using biochemical reconstitution. Various analytical methods have been developed to understand the structure of chromosomes at low to high levels. Here, we introduce a few of them that are commonly used.

### 2.1. Micrococcal Nuclease Assay

**Figure 3:**
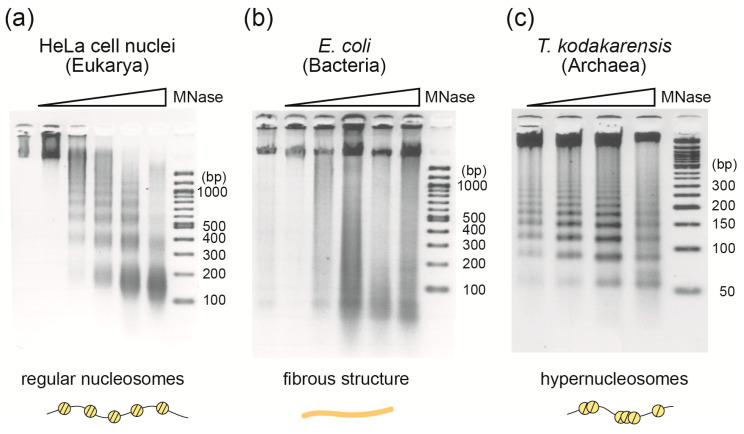
MNase assay revealed different fundamental chromatin structures among the three domains. Different cells from the three domains were treated with MNase in increasing amounts, and the resulting DNA fragments were separated on agarose gels. Schematic representation of the structure implied by each result is shown below the image. (**a**) Nuclei of HeLa cells fixed in 1% formaldehyde; (**b**) *E. coli* cells fixed with 1% formaldehyde; (**c**) Extracted *T. kodakarensis* chromatin. The size of the DNA ladders is shown on the right side of each gel.

Micrococcal nuclease (MNase) is both an endonuclease and an exonuclease and is widely used in the analysis of chromatin structure in eukaryotic cells [3]. When it acts on chromatin, it preferentially digests linker DNA between nucleosomes rather than DNA wrapped around histone octamers in nucleosomes. Digestion of eukaryotic chromatin with MNase, followed by agarose gel electrophoresis, yields a ladder-like pattern of DNA with a minimum of ~150 bp and steps of 150–200 bp, which reflects the length of DNA within the nucleosome core and the regular arrangement of eukaryotic nucleosomes [33] (Figure 3a).

In contrast to eukaryotic chromosomes, digestion of bacterial nucleoids does not result in the protection of DNA of a specific size, neither does it form a ladder-like pattern of protected DNA sizes [34] (Figure 3b). This means that bacterial nucleoids are not composed of structural units of a particular size such as eukaryotic nucleosomes.

In Archaea, MNase digestion has shown that chromosomal DNA of Euryarchaeota is wrapped around DNA-binding proteins (histone or HTa) [35,36,37] (Figure 3c), whereas the chromosomal DNA of Crenarchaeota is not [31]. By combining MNase digestion with high-throughput DNA sequencing, we discovered a unique hypernucleosomal structure from euryarchaea that encodes histones [35] (see Section 3.1).

### 2.2. Mass Spectrometry

Mass spectrometry allows for the identification of proteins in a sample. By combining chromosome isolation, protein separation by SDS-PAGE, and mass spectrometry, chromosome-associated proteins can be identified along with their mass ratios. The advantage of this method is that it enables the discovery of novel chromosomal proteins without relying on homology to other known proteins. For example, chromatin purification, followed by SDS-PAGE and MS identified a novel, abundant chromosomal protein, TrmBL2, in *T. kodakarensis* [27]. In fact, a careful examination of earlier proteomics data of *Thermococcus gammatorelans* cells confirmed the outstanding intracellular abundance of TrmBL2 compared to other normal transcription factors [38]. Recently, a systematic approach was developed for identifying chromosomal proteins based on their abundance, as measured by quantitative mass spectrometry [39]. In eukaryotes, proteomics of sonication-resistant heterochromatin fractions has successfully identified novel proteins involved in heterochromatin formation [40]. Thus, MS is an essential technique for identifying chromosomal proteins, especially those that are difficult to predict based on their similarity to known proteins. This is particularly beneficial when studying archaea, in which a wide variety of chromosomal proteins have yet to be discovered, and in eukaryotes, which have complex chromosome-folding mechanisms that are thought to involve many unknown factors.

### 2.3. Atomic Force Microscopy

As a top-down approach, an on-substrate cell lysis method was developed. In this method, cells attached to a substrate (such as a thin cover glass) are treated with a mild detergent to achieve cell lysis. The combination of this on-substrate lysis with atomic force microscopy (AFM) provides a powerful tool for visualizing and analyzing three-dimensional chromosome structures that are exposed or spread out from the cells. Using this method, hierarchical chromosome structures characteristic of eukaryotic cells [41], bacteria [15,16], archaea [27,31], and chloroplasts [13] have been revealed.

In vitro chromatin reconstitution is a powerful bottom-up approach. In this method, naked DNA is first mixed with chromosomal protein(s) (e.g., histones in the case of eukaryotes) at a relatively high salt concentration, and then the salt concentration is gradually reduced to physiological levels by dialysis [42]. The resulting chromosome structure can be analyzed using AFM [32] or biochemically using a micrococcal nuclease (MNase) assay [43]. The reconstituted chromosomes can be further mixed with external factors to analyze their effects. For example, AFM has shown that the linker histone H1 induces the formation of a 30 nm higher-order chromatin fiber, and that topoisomerase II promotes H1-dependent chromatin compaction [32,44]. Interestingly, archaeal chromatin structure can be reconstituted without salt dialysis and simply by mixing DNA and histones [27]. A combination of chromatin reconstitution and magnetic tweezers has demonstrated competition between archaeal histones and TrmBL2, an abundant chromosomal protein with a transcription factor-like wHTH DNA-binding motif [45].

Reconstitution of eukaryotic chromosome structures beyond the level of eukaryotic 30 nm fiber has not been successful to date. This may be due to the fact of their complexity and the need for a real cellular environment. Recently, a more sophisticated in vitro approach has been proposed. It involves assembling chromosomes using deproteinized genomic DNA and purified DNA organizing elements (such as proteins), followed by encapsulation into cell-sized containers using microfluidics [46]. The application of such an approach would allow for the reconstitution of hierarchical chromosome structures beyond 30 nm fibers.

### 2.4. Chromosome Conformation Capture (3C) and Hi-C

**Figure 4:**
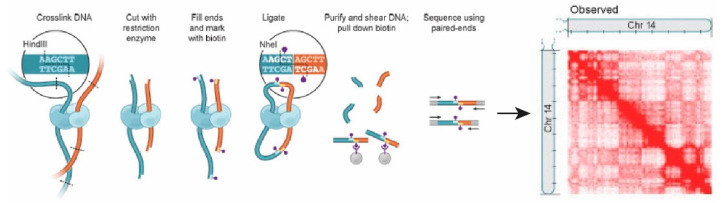
Overview of the Hi-C experiment. Crosslinking of cells with formaldehyde results in covalent attachment of DNA-binding proteins bound to spatially adjacent genomic regions. Chromatin is cleaved with a restriction enzyme (e.g., HindIII, and the single-stranded overhangs created are filled with biotinylated nucleotides (purple dots). The ends of DNA are ligated at low concentrations for intramolecular ligation. DNA is purified and sheared, and streptavidin beads are used to isolate DNA fragments containing biotin label. The interacting genomic DNA regions are identified by paired-end sequencing. Heatmap corresponding to intrachromosomal interactions on chromosome 14 is shown. These figures are adapted from van Berkum et al. [56].

Chromosome conformation capture (3C) technology and its derivatives, such as Hi-C, have enabled the detection of three-dimensional chromosome organization by analyzing the interactions between chromosomal regions inside the cell [47]. In these techniques, genomic DNA is cross-linked, digested with restriction enzymes, and then ligated to quantify the number of interactions between genomic loci that are close together in three-dimensional space, but could be far apart in the linear genome [48] (Figure 4). 3C quantifies the number of interactions between a single pair of genomic loci, whereas Hi-C uses high-throughput sequencing to identify all possible pairs of interactions [48]. Hi-C analysis of eukaryotic chromosomes revealed the presence of subchromosomal compartments, termed A and B. The A compartment is transcriptionally active, whereas the B compartment is transcriptionally inactive [47]. Hi-C has also led to the identification of smaller chromosomal domains, termed topologically associating domains (TADs), within the larger A and B compartments [47,49]. Links between nuclear architecture and pathogenicity of pathogens, such as *Plasmodium* species, a causative agent of human malaria, have also been shown by Hi-C analysis [50]. Single-cell Hi-C has been increasingly being used to map cell-to-cell variability in 3D chromatin structuresstructure in diverse contexts andcontext and suggests that interdomain contacts vary among single cells [51], although computational tools to interpret the data at high resolution are still under development [52,53]. The combination of single-cell and single-molecule techniques is expected to reveal high-resolution chromosome structure and detailed modes of action of the proteins involved in their regulation in the three domains.

Hi-C techniques have revealed that, similar to eukaryotic chromosomes, bacterial nucleoids are also segmented into highly self-interacting regions called chromosomal interaction domains (CIDs), and CIDs are further organized into macrodomains [54]. Hi-C has also been applied to archaeal chromosome structure and revealed that the crenarchaeal chromosome is organized in compartments similar to eukaryotic TADs, and this compartmentalization is dependent on an SMC-family protein [55].

## 3. Fundamental Chromosomal/Nucleoid Proteins

The three domains clearly diverged in how they organize their chromosome structures. However, there are proteins that are commonly used in more than one domain such as histone, HU, and SMC proteins. In this section, we focus on the roles of individual chromosomal proteins in understanding the principles of chromosome folding.

### 3.1. Histone

Histones are the most fundamental building blocks of eukaryotic chromosomes. In 1990, the first archaeal histone was discovered in *Methanothermus fervidus*, a methanogenic archaeon [57]. Later, it became evident that species in Euryarchaeota (a major phylum in Archaea), except for *Thermoplasmata*, encode proteins homologous to eukaryotic histones. Species in other phyla, such as Nanoarchaeota, Thaumarchaeota, and Lokiarchaeota, also encode histones [58]. Archaeal histones are smaller (~6 kDa) than eukaryotic histones, and they do not possess the tail region that undergoes post-translational modification and is involved in the regulation of chromosome structure and gene expression in eukaryotes [59]. A recent report of lysine-specific acetylated proteome from *T. gammatolerans* suggests that archaeal histones undergo acetylation [60]. However, it needs to be clarified whether this modification really takes place in the cell and has a physiological role. Phylogenetic analysis indicates that there are two paralogues of archaeal histones: histone A and histone B. In contrast to the strict subunit composition of eukaryotic histones (a histone octamer consisting of an H3/H4 tetramer with two H2A/H2B dimers), which is defined by the structure of the four-helix bundle (4HB), archaeal histones can form either homo- or hetero-dimers [58,59]. Studies using AFM have shown that the size of a single archaeal “nucleosome” is approximately 9 nm [27] (Figure 1).

Based on early crosslinking and electron microscopic studies, archaeal histones exist as dimers in solution and as stable tetramers in the presence of DNA [30]. However, careful observation of the MNase-digested pattern of DNA from *T. kodakarensis* (a minimum of 60 bp fragment and a ladder of 30 bp steps) suggested the presence of structures other than the histone tetramer (Figure 3c). This is because if archaeal chromatin is a simple beads-on-a-string structure formed by nucleosomes consisting only of histone tetramers and the linker DNA between them, the size of MNase-protected DNA would be a multiple of 60 bp, without the 30 bp steps that were actually observed [27]. 

In 2013, analysis of DNA sequence associated with the MNase-protected ladder showed that archaeal ”nucleosome” is not restricted to tetramer, but it is a flexible structure composed of histone dimer units that can pile up to form larger complex [35]. In these structures, the histone dimer is a unit that can bind ~30 bp of DNA. It was proposed that a unit of archaeal histone dimer can stack on each other using its flexible 4HB domain, resulting in the formation of an archaea-specific nucleosome that is flexible in its number of histone dimer units [35].

Since the first proposal of an alternative chromatin structure model specific to archaea [35], a number of experimental studies have proved its correctness. For example, crystallographic analysis of archaeal nucleosomes revealed that certain amino acids within the 4HB domains are responsible for the multiplex stacking of dimers; mutations in these amino acids resulted in the loss of the multiplex ladder pattern in the MNase assay [36]. These mutations have led to changes in the expression of several genes [36], but the biological significance of this structure is not yet fully understood. The flexible multimeric structures formed with archaeal histone have been assigned various names including ”hypernucleosome” [58], ”archaeasome” [61], and ”archaeal histone-based chromatin polymers (AHCP)” [62]. In this review, the term ”hypernucleosome” was adopted.

The structure of the hypernucleosome may dynamically change depending on the state of the cell and the environment, thereby regulating gene expression in response to changes in the environment [36,62]. Another important question is the extent to which hypernucleosomes are present on chromosomes in living cells. Furthermore, since hypernucleosomes are not found in all archaeal species [63], it is possible that they play a role in the evolution of certain lineages of archaea. 

Interestingly, although a majority of eukaryotic nucleosomes contain regular histone octamers, several types of non-canonical nucleosomes exist. For example, histone H3 is replaced with the centromere-specific histone CENP-A in eukaryotic centromeres [64]. Moreover, other structural variants of the nucleosome have been found such as overlapping dinucleosomes, which are formed by nucleosome collision during chromatin remodeling, and hexasomes, which are formed by removing one H2A/H2B dimer from a dinucleosome, that are formed during the transcription process [64]. The discovery of a flexible hypernucleosome structure in archaea raises the possibility that the archaeal ancestor of eukaryotes already had the ability to regulate chromatin structure through modulation of histone-based chromatin structure [59].

### 3.2. Alba

Alba is a 10-kDa DNA/RNA-binding protein found widely in archaea including Euryarchaeota, Crenarchaeota, and newly proposed phyla such as Nanoarchaeota, Korarchaeota, Thaumarchaeota, and Lokiarchaeota [30]. Alba undergoes post-translational modifications, including acetylation and methylation [65], and has several different modes of interaction with DNA such as DNA stiffening or bridging [66]. AFM revealed that Alba forms a ~10 nm fiber without wrapping DNA [27,31].

A comparison of chromosome structures of different archaeal lineages using a combination of in-vitro reconstitution and AFM revealed that Alba-mediated chromosome structures may differ depending on the type of other chromosomal proteins expressed in the cell [31]. For example, Alba and histone are co-expressed in several archaeal lineages, including some euryarchaea [65]. In-vitro reconstitution and competition experiments combined with AFM showed that increasing the amount of Alba decreases the extent of DNA wrapping by histones [31]. This suggests that Alba plays a role in regulating transcription and chromosome structure by modulating the extent of DNA topology determined by histone binding.

Alba superfamily proteins are also found in eukaryotes and have been suggested to be involved in RNA metabolism [65]. It remains to be seen whether eukaryotic Alba plays a role in genome folding. 

### 3.3. HU

HU is a major bacterial NAP that bends DNA. AFM analysis of lysed *E. coli* cells has shown that its nucleoid is composed of 30–80 nm structures, and RNase A degraded the size of the 30–80 nm structures into 10 nm fibers, indicating that RNA is involved in the formation of the 30–80 nm higher-order structure, but not in the 10 nm fiber [12]. AFM analysis of nucleoid fibers derived from *E. coli* strains that lack a single NAP gene indicates that there is no single NAP that is responsible for the formation of the 10 nm fiber. However, it is clear that proteins (NAPs or topoisomerase) are involved in the formation of the 10 nm fiber, because naked DNA appears only after proteinase K treatment of the nucleoid [67]. 

HU protein is associated with the survival of some bacterial species and regulates a variety of cellular processes, such as growth and SOS response [68]. Single-molecule tracking has shown that HU has several different modes of DNA binding and indicated that HU plays a dual function of compacting the nucleoid through specific DNA structure-binding and decondenses the nucleoid through nonspecific and weak interactions with the genomic DNA [69].

In the case of archaea, species in Thermoplasmatales are unique in that, although they belong to Euryarchaeota, they lack histones. Instead, they encode HTa, a protein homologous to the bacterial HU [37]. Early studies have shown that HTa is associated with *Thermoplasma* genomic DNA and protects about 40 bp of DNA at a minimum [70]. Although phylogenetic analysis suggests that HTa was horizontally transferred from bacteria to archaea at some point [37], it remains unclear whether HTa plays the same role as bacterial HU (i.e., to bend DNA). Several recent studies on HTa have shown that, unlike bacterial HU, HTa in *Thermoplasma* wraps DNA, forming particles of approximately 6 nm [31,37] (Figure 1). Therefore, DNA wrapping seems to be a common requirement for DNA folding in Euryarcaheaota, regardless of the protein used (histone or HTa).

We propose that, at some point after the horizontal transfer of the bacterial HU to the ancestor of *Thermoplasma*, HTa acquired the ability to wrap DNA; when and how this shift occurred should be a topic for future studies. After HTa adapted to the archaeal environment, including acquiring the ability to wrap DNA, the histone gene has been lost. For the chromosomes of Euryarchaeota, DNA wrappers may be indispensable for maintaining or relaxing the topological state of DNA, or for other unknown reasons. Therefore, Euryarchaea must encode a protein that fulfills this role, but that protein does not necessarily have to be a histone.

### 3.4. Suppression of Horizontally Transferred Genes by Global Regulatory Proteins

Horizontal gene transfer (HGT) is fundamental to archaeal and bacterial evolution [71,72]. Various mechanisms of gene flow in archaea have been revealed, for example, transformation in Euryarchaeota, vesicle transport in Thermococcales, transduction (via virus), conjugation in *Sulfolobus*, cell fusion in Haloarchaea, and chromosomal DNA exchange in Crenarchaea [71,72].

H-NS is an NAP that functions as a global repressor of transcription in bacteria. It preferentially binds to AT-rich sequences and can bridge two DNA strands together [11]. H-NS can suppress the expression of downstream genes by binding to the promoter region and forming a filamentous structure or forming loops [73]. This characteristic of H-NS is involved in virulence of some bacteria, as well as in the suppression of horizontally transferred genes, a function called ”xenogeneic silencing” [74]. When a foreign DNA element is incorporated into the genome, H-NS covers the region, thereby suppressing the expression of potentially harmful genes. H-NS detects foreign DNA by differences in GC content compared to the host genomes [74]. This is important in driving prokaryotic evolution by promoting the horizontal transfer of genes while suppressing their toxic effects [75]. Although HGT frequently occurs between archaea and bacteria, such xenogeneic silencing was not known in archaea.

**Figure 5:**
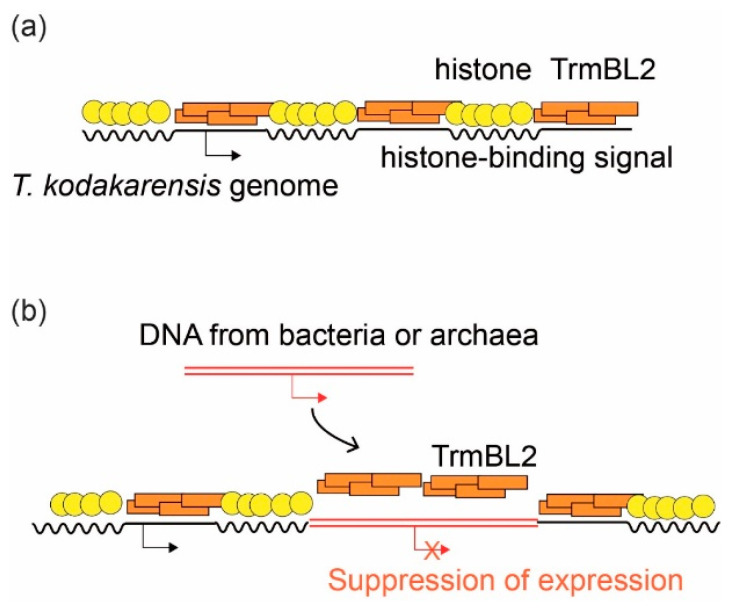
A model of how the transcription factor-like chromosomal protein TrmBL2 represses the expression of horizontally transferred genes in archaea. The chromosome of *T. kodakarensis* chromosome is shown as an example. (**a**) The genomic sequence and the state of chromosomal protein binding. Histone localization is determined by the histone binding signals on genomic DNA, and TrmBL2 bind to genomic regions with low histone localization because of its low sequence specificity. (**b**) When foreign DNA is obtained from bacteria, TrmBL2 essentially covers the entire region, because bacterial DNA does not have a histone-binding signal. This way, the expression of potentially harmful genes is repressed in the early stages of horizontal gene transfer.

Recently, a model was proposed that the archaeal chromosomal protein TrmBL2 plays a role similar to that of H-NS in suppressing horizontally transferred genes [76]. TrmBL2 is a transcription factor-like protein with a helix-turn-helix DNA-binding motif, which was initially identified as an abundant chromosomal protein that forms a thick (~14 nm) nucleoprotein filament on DNA without shortening (wrapping) DNA. Interestingly, TrmBL2 binds to both coding and non-coding regions and suppresses gene expression when bound to the promoter region [27]. In contrast to TrmBL2, which can bind to genomic DNA without sequence specificity, the (archaeal) histone binding site on the genomic DNA is more strictly defined by the histone-positioning signal [35,77]. Single-molecule analysis using magnetic tweezers revealed that the TrmBL2 protein of *T. kodakarensis* competes with archaeal histones for DNA binding [45]. Therefore, TrmBL2 may be located where there is no histone positioning signal on the DNA, and when the binding happens to be in the promoter region, the gene expression is suppressed. Indeed, histone frequency is lower in the promoter region of *T. kodakarensis* [35,77]. We propose that genes on DNA segments that are horizontally transferred from bacteria or non-histone-coding archaea can be suppressed by TrmBL2 in this manner (Figure 5). These facts support the idea that TrmBL2 is indeed a missing xenogeneic silencer in archaea.

As discussed above, proteins that serve as transcriptional repressors and genome folding factors also play an important role in suppressing horizontally transferred genes. Are there any such silencer proteins in eukaryotes? The horizontal transfer of genes from bacteria to eukaryotes is known, and several mechanisms have been proposed for how the expression of these genes is initially regulated, but it is not assumed that transcriptional repressors are involved [78]. In fact, to the best of our knowledge, general xenogeneic silencers such as H-NS in bacteria and TrmBL2 in archaea are absent in eukaryotes. Since eukaryotes have a different gene structure from archaea and bacteria (e.g., eukayotes have introns), they may handle this problem in a way that is different from that of prokaryotes [78]. In summary, the coupling of chromatin structural proteins and the silencing of horizontally transferred genes may be unique to archaea and bacteria.

### 3.5. SMC Proteins Are Involved in 3D Structure Formation in the Three Domains of Life

**Figure 6:**
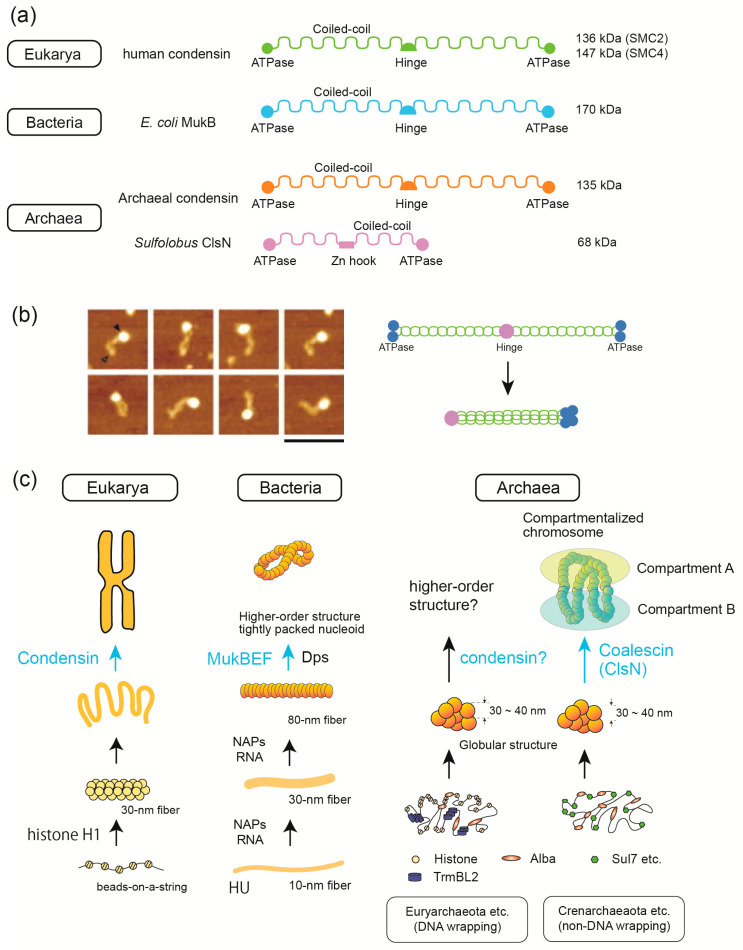
SMC proteins are commonly involved in 3D structure formation in the three domains of life. (**a**) Comparison of SMC proteins in the three domains. Their domain structures and molecular weight are shown. (**b**) AFM images of fission yeast condensin heterodimers with a head–tail structure (left). The head (filled triangle) consists of four globular ATPase domains, and the tip of the tail (open triangle) represents the hinge regions. Scale bar, 100 nm. As shown in the hypothetical model (right), the coiled-coil region (green) is folded back at the hinge (purple), and four globular domains (blue) are assembled. The AFM images were taken from Yoshimura et al. [80]. (**c**) Although various proteins are involved in genome folding at the basic level, SMC proteins (marked in light blue) are commonly involved in the highest level of folding. In eukaryotes, the beads-on-a-string structure folds into 30 nm fibers with the help of linker histone H1. Condensin contributes to the formation of mitotic chromosomes. In bacteria, conserved HU forms 10 nm fibers, and then other NAPs and various types of RNAs contribute to the formation of 30 and 80 nm fibers; MukBEF (SMC complex in *E. coli*) is involved in higher-order structuring of nucleoid; Dps is responsible with stress-induced compaction. In archaea, regardless of the mode of basic folding of the genome (either DNA wrapping in Euryarchaeota or non-DNA wrapping in Crenarchaeota), the genomes are commonly folded into 30–40 nm globular structures [31]. In Crenarchaeota, the SMC protein ClsN is implicated in chromosome compartmentalization. It needs to be elucidated as to if SMC proteins play a similar role in Euryarchaeota.

SMC family proteins are essential for higher-order chromosome folding in the three domains of life. In general, SMC proteins are composed of ATPase domains, coiled-coil regions, and a hinge domain (Figure 6a). SMC proteins usually form larger functional complexes with accessory proteins [7]. In eukaryotes, condensin and cohesin complexes play roles in mitotic chromosome condensation and sister chromatid cohesion, respectively [7]. Smc5/6 complex plays a role in the cellular response to DNA damage [79]. Single-molecule analysis using AFM revealed that condensin heterodimer forms a head–tail structure and that the ATPase activity of condensin is regulated by the binding of non-SMC trimer to the head of the SMC heterodimer [80] (Figure 6b). AFM also showed that condensin but not cohesin induces DNA reannealing through protein–protein assembly [81]. Loop extrusion by condensin and cohesin has been proposed as a mechanism underlying their function in genome organization [82]. Detailed analysis using protein engineering and mutagenesis in conjugation with single-molecule experiments revealed that loop extrusion depends on five DNA binding sites in the case of cohesin [83]. Single-particle tracking using photo activated localization microscopy in live fission yeast revealed that Smc5/6 localize in the nucleus throughout the cell cycle but exhibited more dynamic association with chromatin compared with cohesin [79].

Bacterial species encode proteins homologous to eukaryotic SMCs. Bacterial SMCs have a domain structure similar to that of eukaryotic condensin and cohesin (Figure 6a), and they play a role in bacterial nucleoid organization. For example, MukBEF in *E. coli* and Smc-ScpAB in *Bacillus subtitles* are involved in the formation of higher-order nucleoid structures [84] (Figure 6c). Single-molecule tracking of *B. subtilis* nucleoids revealed that SMC operates by different patterns of motion compared to other DNA-condensing NAPs such as gyrase and HBsu (an HU family protein) [85,86]. Electron cryomicroscopy (cryo-EM) single-particle analysis revealed the detailed mechanism of how *E. coli* MukBEF entraps two distinct DNA strands when bound to the unloader MatP [87].

The condensin complex is highly conserved across all three domains of life and plays an important role in higher-order chromosome folding [88] (Figure 6c). Although proteins homologous to condensin are found in archaea, *Sulfolobus* species lack condensin. Recently, coalescin (ClsN), a novel *Sulfolobus*-encoded SMC protein, was shown to be involved in the organization of chromosomes into a two-domain compartment structure resembling the eukaryotic A/B compartments [55]. ClsN is shorter than condensins and has a Zn hock in the middle as in the case of eukaryotic Rad50 (Figure 6a). Comparison of ClsN binding (ChIP-seq) and transcriptional activity (RNA-seq and RNA polymerase localization) indicated that ClsN is associated with the transcriptionally inactive B-compartment [55].

Hi-C analysis of euryarchaeal chromosomes showed slightly different structures. The chromosome of *Haloverax volcanii*, an extreme halophilic euryarchaeon, and *T. kodakarensis*, contains multiple large chromatin loops and self-interacting domains similar to bacteria and eukaryotes. Unlike *Sulfolobus* species, these euryarchaeal chromosomes are not organized in a way that separates transcriptionally active and inactive compartments [89]. It remains to be elucidated whether there is a general rule for chromosome compartmentalization in archaea, the proteins responsible for higher-order genome folding in each archaeal lineage, and the physiological roles of these higher-order structures.

Structurally diverse SMC proteins were found to play similar roles in the higher-order genome folding in the three domains. Interestingly, the absence eukaryotic condensin-like protein and the use of a lineage-specific SMC (i.e., ClsN) coincides with the absence of DNA wrapping at a fundamental level of genome folding in *Sulfolobus*. Investigating this association between fundamental and higher-order chromosome structures in *Sulfolobus* and comparing it to other lineages of life may provide clues as to how different genome folding mechanisms have evolved.

## 4. Conclusions

Many studies on chromosome structure have been obtained from studies on eukaryotes and bacteria, and new insights are coming from archaeal chromosome studies. Further studies may contribute to the understanding of the general principles of genome folding across the three domains. Comparative structural/molecular biology using single molecule/cell techniques has been successful and revealed hierarchic structures in all three domains of life. Histone, Alba, and HU are the main proteins that build-up the initial steps for this hierarchy in Eukarya, Archaea, and Bacteria, respectively. At this level, bacterial H-NS and archaeal TrmBL2 are possibly domain-specific global regulatory proteins that also function as suppressors of horizontally acquired genes. SMC proteins, such as condensin (Eukarya), MukBEF (Bacteria), and ClsN (Archaea), are involved in the later stages of the construction of higher-order chromosomal/nucleoid structures. At present, little is known about how intermediate levels of hierarchical genome folding are achieved in these three domains. Further interesting information on genome-folding mechanisms will be obtained from single-molecule and single-cell analyses.

## Data Availability

Not applicable.
